# Proton Exchange Magnetic Resonance Imaging: Current and Future Applications in Psychiatric Research

**DOI:** 10.3389/fpsyt.2020.532606

**Published:** 2020-09-24

**Authors:** Joseph J. Shaffer, Merry Mani, Samantha L. Schmitz, Jia Xu, Nana Owusu, Dee Wu, Vincent A. Magnotta, John A. Wemmie

**Affiliations:** ^1^Department of Radiology, University of Iowa, Iowa City, IA, United States; ^2^Department of Psychiatry, University of Iowa, Iowa City, IA, United States; ^3^Pappajohn Biomedical Institute, University of Iowa, Iowa City, IA, United States; ^4^Department of Biomedical Engineering, University of Iowa, Iowa City, IA, United States; ^5^Department of Radiology, University of Oklahoma Health Sciences Center, Oklahoma City, OK, United States; ^6^Department of Veterans Affairs Medical Center, Iowa City, IA, United States; ^7^Department of Molecular Physiology and Biophysics, University of Iowa, Iowa City, IA, United States; ^8^Department of Neurosurgery, University of Iowa, Iowa City, IA, United States

**Keywords:** neuroimaging, T1ρ MRI, chemical exchange saturation transfer (CEST) imaging, psychiatric disorders and mental health, proton exchange

## Abstract

Proton exchange provides a powerful contrast mechanism for magnetic resonance imaging (MRI). MRI techniques sensitive to proton exchange provide new opportunities to map, with high spatial and temporal resolution, compounds important for brain metabolism and function. Two such techniques, chemical exchange saturation transfer (CEST) and T1 relaxation in the rotating frame (T1ρ), are emerging as promising tools in the study of neurological and psychiatric illnesses to study brain metabolism. This review describes proton exchange for non-experts, highlights the current status of proton-exchange MRI, and presents advantages and drawbacks of these techniques compared to more traditional methods of imaging brain metabolism, including positron emission tomography (PET) and MR spectroscopy (MRS). Finally, this review highlights new frontiers for the use of CEST and T1ρ in brain research.

## Introduction

Unraveling the biochemical signatures of cellular metabolism and neuronal activity is critical, not only for our basic understanding of brain function, but also for understanding neurological and psychiatric disorders. Positron emission tomography (PET)-based methods and magnetic resonance spectroscopy (MRS) have been established as proven techniques both for clinical routine and investigational neuroscience studies to measure brain metabolism. In this review, we examine two MRI techniques that offer additional possibilities for imaging brain metabolism by tapping into a physical phenomenon known as proton exchange. These MRI techniques are named chemical exchange saturation transfer (CEST), and T1 relaxation in the rotating frame (T1ρ). Both methods provide whole-brain coverage with high resolution, can be used to monitor pH changes in brain tissues, as well as to measure and map various brain metabolites and neurotransmitters. These techniques provide complementary information to MRS and PET and avoid some of the drawbacks of these techniques. Therefore, proton-exchange imaging may provide useful approaches to address certain research questions and may also hold great potential for clinical applications.

The purpose of this review is threefold. First, we seek to simplify the MRI physics underlying these methods in order to make them understandable to non-experts. Second, we review their current status and use in neuroscience research. Finally, we discuss their potential use to help achieve a better understanding of brain metabolites and neurotransmitters.

## Proton Exchange

**Figure 1:**
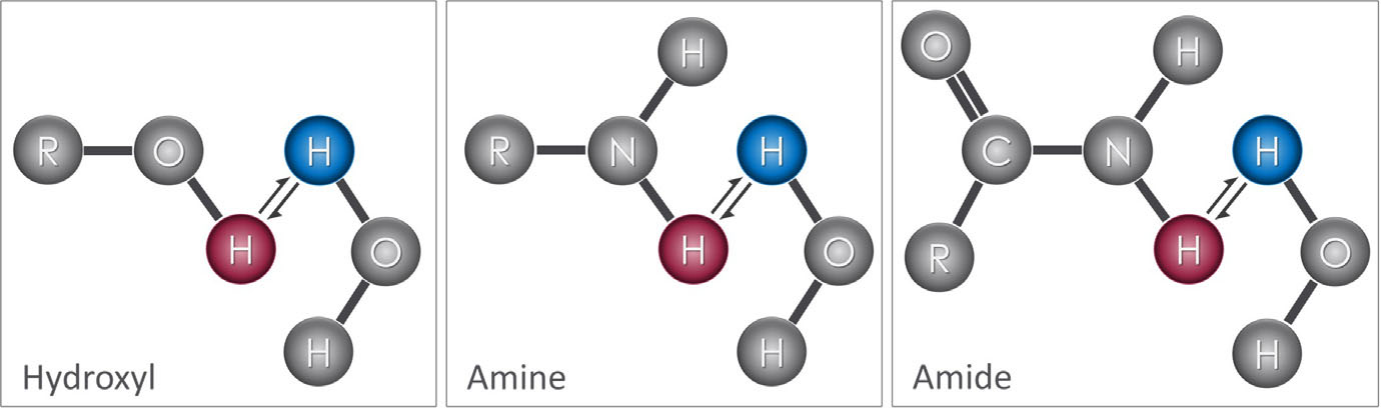
Proton exchange: Hydroxyl, amine, and amide functional groups attached to molecules contain exchangeable protons (red) that can be readily exchanged with protons in water (blue). When exchange occurs, the proton that was originally part of the solute molecule is attached to the water molecule and vice-versa. This exchange process occurs continuously, transferring protons between bulk water and solute molecules.

Proton exchange (**Figure 1**) is a well-known phenomenon in which protons from the bulk water are exchanged with labile protons from soluble molecules (e.g. metabolites and neurotransmitters). MR imaging techniques have been developed that capitalize on the proton exchange phenomenon to indirectly provide information about the concentrations of these molecules. To better understand proton-exchange imaging, a basic overview of MR physics used in traditional MR imaging is provided in **Box 1** below for non-MRI experts.

BOX 1 **Basic MRI Physics**

**Figure 2:**
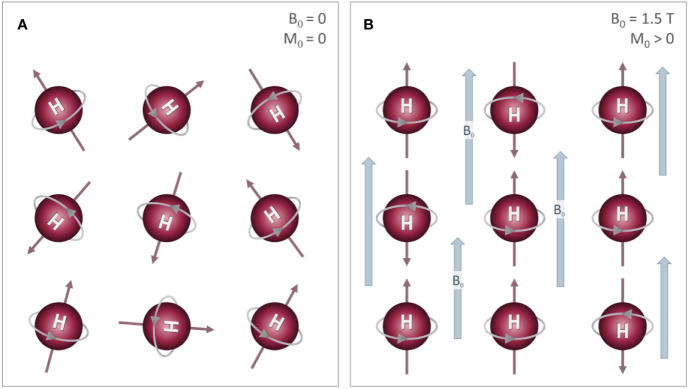
Effects of an external magnetic field on proton alignment: The nucleus of the hydrogen (H) atom contains a single proton that spins on its axis, generating a small magnetic field (i.e. north (N) to south) represented by the red arrows. **(A)** In the absence of an external magnetic field, these are randomly oriented, which results in a net magnetization (M_0_) of zero. **(B)** When these protons are placed inside a strong magnetic field (B_0_), their orientations align either parallel or anti-parallel to the B_0_ field with a slightly more protons aligned parallel to B_0_. This difference between the two alignments results in a small net magnetization (M_0_) that is parallel to B_0_.

**Figure 3:**
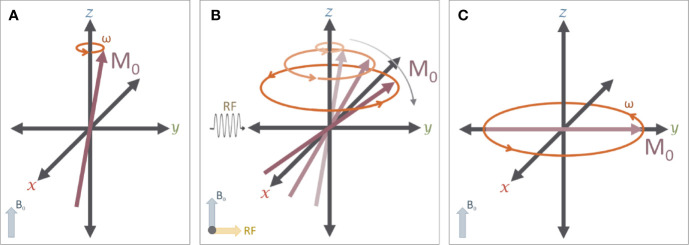
The effects of a RF pulse: **(A)** A typical MR experiment begins with protons in a strong magnetic field (B_0_) that is aligned with the z axis (down the bore of the MR scanner). As noted in **Figure 2**, these protons spin on their axis (grey arrow), generating a small amount of net magnetization (M_0_, red arrow)) that precesses around the z-axis (orange arrow) at the Larmor frequency (ω). **(B)** A 90° radiofrequency (RF) pulse is applied to the system in order to “tip” M_0_ into the transverse (X-Y) plane. The net magnetization M_0_ will exhibit a spiral trajectory from the initial starting orientation as shown. **(C)** Immediately after the RF pulse is removed, the net magnetization (M_0_) is rotating around the z-axis on the transverse plane.

In most MR imaging techniques, the signal comes from hydrogen atoms present in water molecules. The nucleus of the hydrogen atom consists of a single positively charged proton. These protons are highly mobile (i.e. “free protons”) and are sensitive to electrochemical and magnetic forces. The magnetic field of the MR scanner, known as the main magnetic field, causes these protons to spin about their axes in specific orientations, either parallel or antiparallel to the main magnetic field (**Figure 2**). The parallel alignment has a lower energy state causing a slight majority to align in this orientation and producing a net magnetization known as the longitudinal magnetization (M_0_). M_0_ precesses around the main magnetic field at a specific frequency, known as the Larmor frequency (ω), which is proportional to the main magnetic field strength (B_0_) according to the following equation:

(1) $$\omega =\gamma B_{0}$$

where γ is the gyromagnetic ratio, a constant that is specific to the nucleus of interest (42.577 Mhz/T for ^1^H) (**Figure 3A**). Applying radiofrequency (RF) pulses at the Larmor frequency tips the net magnetization away from main field (**Figure 3B**) with an angle determined by the product of the RF amplitude and duration.

**Figure 4:**
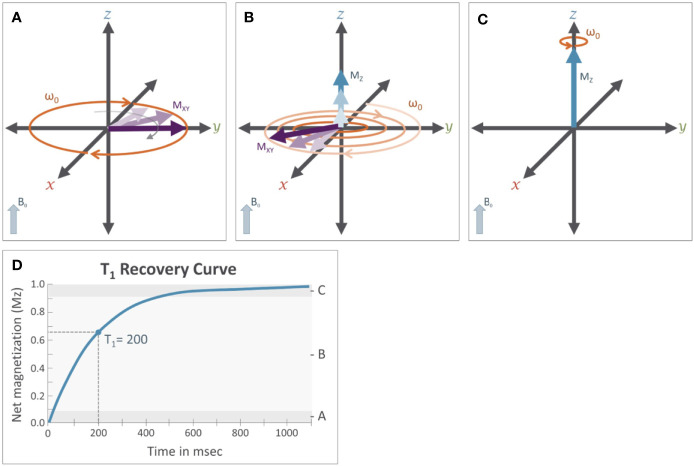
T1 relaxation: M_0_ is the initial longitudinal magnetization (M_z_=M_0_) before the application of the RF pulse. M_0_ is tipped into the transverse plane by the application of the 90° RF pulse (M_z_=0). Immediately after removal of the 90° RF pulse (see **Figure 3C**) **(A)**, magnetization will recover from the transverse plane. While this is occurring **(B)**, the net magnetization along the z-axis (M_z_, blue arrows) will increase while transverse magnetization (M_xy_, purple arrows) will decrease until the net magnetization returns to its original orientation **(C)** aligned with the B0 field (M_z_ = M_0_). The magnitude of the net magnetization along the Z axis follows an exponential recovery curve **(D)**, with T1 relaxation time being the amount of time it takes for magnetization on the z-axis to return to M_z_.

In traditional MR imaging (e.g. T1- and T2-weighted imaging), RF pulses tip the net magnetization into the transverse plane (**Figure 3C**). Once the RF pulse is turned off, the spins and corresponding transverse magnetization (M_xy_) produces measurable signals, which are influenced by two relaxation properties (T1 and T2 relaxation). T1 relaxation is the process by which the net magnetization returns to alignment with the main magnetic field (**Figure 4**). Assuming that the initial magnetization in the longitudinal plane is M_0_ and a 90 degree RF pulse is applied that tips M_0_ to the transverse plane, T1 is defined as a time constant whereby the longitudinal component of the magnetization (M_z_) recovers exponentially with time (t). The recovery of the magnetization along the main magnetic field over time is characterized by the following equation:

(2) Mz(t)=M0∗(1−e−tT1)

Here, T1 is the time it takes for M_z_ to return to approximately 63% of the initial magnetization (M_0_) in the longitudinal plane. Because the rate of T1 relaxation depends on the tissue structure (e.g. lattice) it is often referred to as “spin-lattice” relaxation and provides an important source of contrast across different types of tissue. For example, in cerebrospinal fluid (CSF) protons are unable to dissipate energy as fast as protons in a more solid tissue, and so they exhibit a longer T1 relaxation time, which ultimately causes CSF to appear darker on an image accentuating T1-dependent contrast, also known as a T1-weighted image.

The T2 relaxation process is produced by local magnetic field variations (δ_i_), which cause subtle differences in Larmor frequencies of individual free protons (ω_i_) determined by Equation 3, which is a simple extension of Equation 1.

(3) $$\omega_{i}=\gamma (B_{0}-\delta_{i})$$

**Figure 5:**
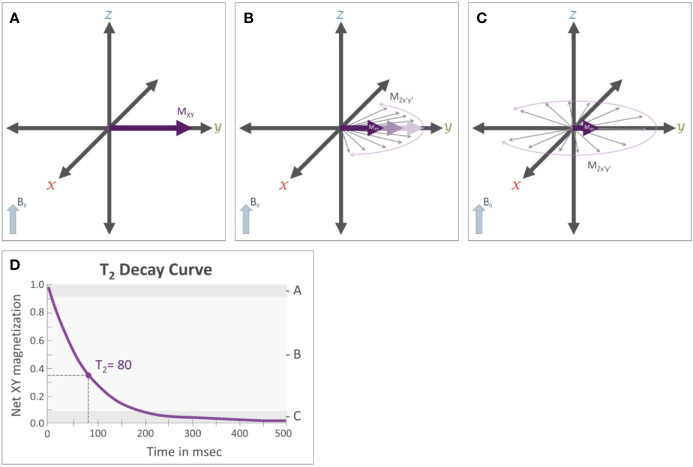
T_2_ relaxation: Immediately after the application of a 90° RF pulse (see **Figure 3C**) **(A)** the net magnetization of the population of protons is rotating along the transverse plane at the Larmor frequency (ω). Importantly, these protons are rotating *in phase* with each other. **(B)** T_2_ relaxation results from subtle variations that occur in the magnetic field that cause slight variation in the Larmor frequency of individual protons. This results in a loss of coherence where some protons are rotating faster while others rotate more slowly; causing the magnetic fields of individual protons, represented by the thin gray arrows, to be slightly out of phase with each other and causing the net magnetization (M_XY_) in the transverse plane to decrease. **(C)** After sufficient time has passed, these individual protons will be distributed randomly along the transverse plane, causing M_xy_ to approach 0. **(D)** This reduction in M_xy_ follows an exponential decay curve and the T2 relaxation time constant.

Local field variations cause the spins to lose their coherence over time, which in turn causes the associated magnetization in the transverse plane to decay (**Figure 5**). The decay of the transverse magnetization is commonly referred to as “spin-spin” relaxation. Assuming a 90° tip angle, the decay of the transverse plane magnetization (M_xy_) over time (t) is defined by Equation 4

(4) Mxy(t)=M0∗(e−tT2)

where T2 is a time constant for the relaxation process. That is, T2 is the time it takes for M_xy_ to fall to approximately 37% of its initial value (M_0_). Using CSF again as an example, the local magnetic field variability is relatively low in CSF as compared to other brain tissues and thus has a long T2 relaxation time. Therefore, CSF appears bright on T2-weighted images relative to gray matter and white matter, which have greater local magnetic field variations.

As explained above, traditional T1- and T2-weighted imaging capitalize primarily on the magnetization of protons in the bulk water. However, imaging other molecules with protons can provide useful information regarding brain metabolites and neurotransmitters. Sensitizing MRI measurements to the magnetization from the protons of the other molecules requires different acquisition strategies because they resonate at a different frequency from that of the bulk water protons. Moreover, these molecules occur at much lower concentrations (approximately 10^−5^ times less) than bulk water. One strategy to generate MRI contrast from these soluble molecules is to measure the influence of their exchangeable protons on the larger bulk water pool through the phenomenon of proton exchange. CEST and T1ρ are examples of such imaging techniques.

## Proton Exchange Imaging

Several compounds contain protons that are readily available for proton exchange (**Figure 1**). Such exchangeable protons are commonly found on amine (-NH_2_), amide (-NH), thiol (-SH), and hydroxyl (-OH) groups, that are present in a wide variety of molecules including various brain metabolites and neurotransmitters. However, the protons present in these molecules experience slight variations in their Larmor frequency based on their local electromagnetic environment. On the other hand, protons present in bulk water precess at the Larmor frequency of the main magnetic field. Because water protons and exchangeable protons have different Larmor frequencies, they can be selectively marked, manipulated, and monitored using MR imaging techniques.

The difference in Larmor frequency between exchangeable solute protons (ω_s_) and free protons (ω_0_) is denoted by δω_s_ = ω_s_-ω_0,_ measured in Hz. The frequency difference can also be expressed in parts-per-million (ppm), in which case, the term chemical shift is used to denote the shift between ω_s_ and an absolute reference standard (ω_ref_) normalized to the Larmor frequency of the scanner. RF pulses can be applied at specific Larmor frequencies to selectively manipulate (increase or suppress) the net magnetization of either the water pool or the solute pool. Although the net magnetization of both pools is sensitive to proton exchange, differences in magnetization are easier to measure in the water pool due to its greater abundance. The phenomenon of proton exchange is leveraged by both CEST (1) and T1ρ imaging (2), but in somewhat differing ways. In the following sections, we explore these two techniques in more detail.

## Cest Acquisition and Analysis

**Figure 6:**
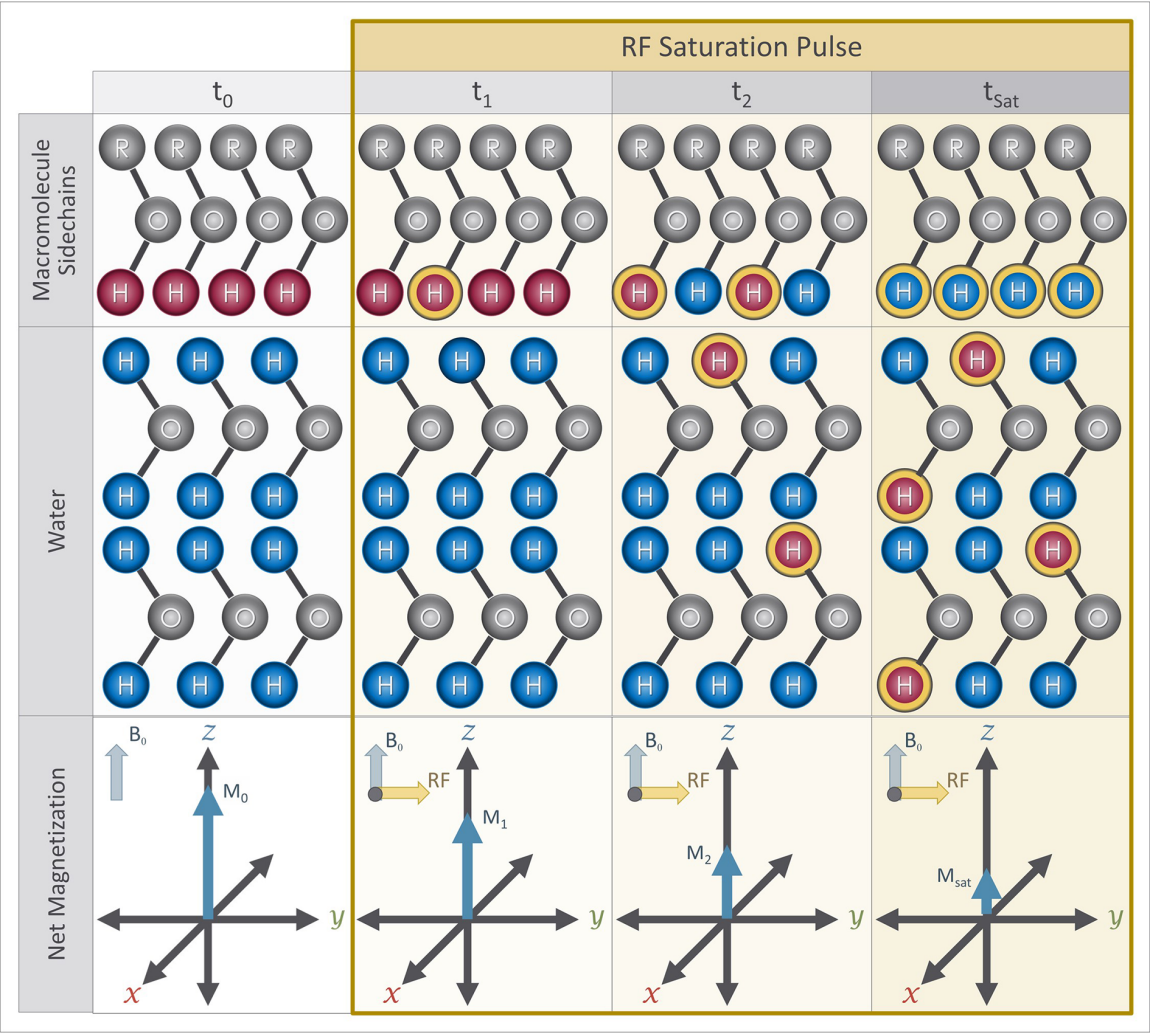
Magnetization preparation blocks for CEST imaging sequence: At the beginning of a CEST experiment, protons attached to the solute molecule of interest (red) have a different Larmor frequency from protons in bulk water (blue). A RF saturation pulse is applied at the Larmor frequency of the exchangeable protons of the solute molecule, which causes some of them to become saturated (yellow outline). Over time, the saturated protons exchange with the unsaturated protons in water due to proton exchange while the saturation pulse continues to saturate protons attached to the solute pool, including protons that were originally in the water pool (blue with yellow outline). The presence of the saturated protons in the water results in a reduction of the net magnetization measured from the water over time. The net magnetization at saturation (M_sat_ ) is therefore lower than the initial net magnetization (i.e. M_sat_ < M_0_).

CEST imaging uses an RF pulse to selectively suppress the magnetization of a desired pool of solute protons. Specifically, a long RF “saturation” pulse (S_sat_) with a narrow bandwidth is applied, that is tuned to match the Larmor frequency of the exchangeable protons (ω_s_) of a solute of interest. The application of the RF saturation pulse suppresses the magnetization of the solute protons (**Figure 6**). These saturated solute protons then exchange with the unsaturated water protons causing a measurable reduction in the magnetization from the water pool. The long duration of the saturation pulse allows enough time for the effect of the saturation to reach steady state. This reduction in magnetization can be used to calculate the concentration of the molecule of interest.

One would ideally want the saturation pulse to only influence the solute protons of interest. However, the applied saturation pulse has some direct saturation effects on the water pool as well as a broad effect on other molecules. To minimize the undesired effects of the saturation pulse, a minimum of three measurements are performed where saturation pulses are applied symmetrically with respect to the water frequency, at +δω_s_ and -δω_s,_ as well as a measurement without any saturation pulse (S_0_). The two images collected with saturations pulses are subtracted and normalized by the signal acquired without any saturation pulse. This is known as the CEST asymmetry ratio (CEST_asym_)

(5) $$CEST_{asym}(\omega_{s})=\frac{S_{sat}(-\Delta \omega_{s})-S_{sat}(+\Delta \omega_{s})}{S_{0}}$$

**Figure 7:**
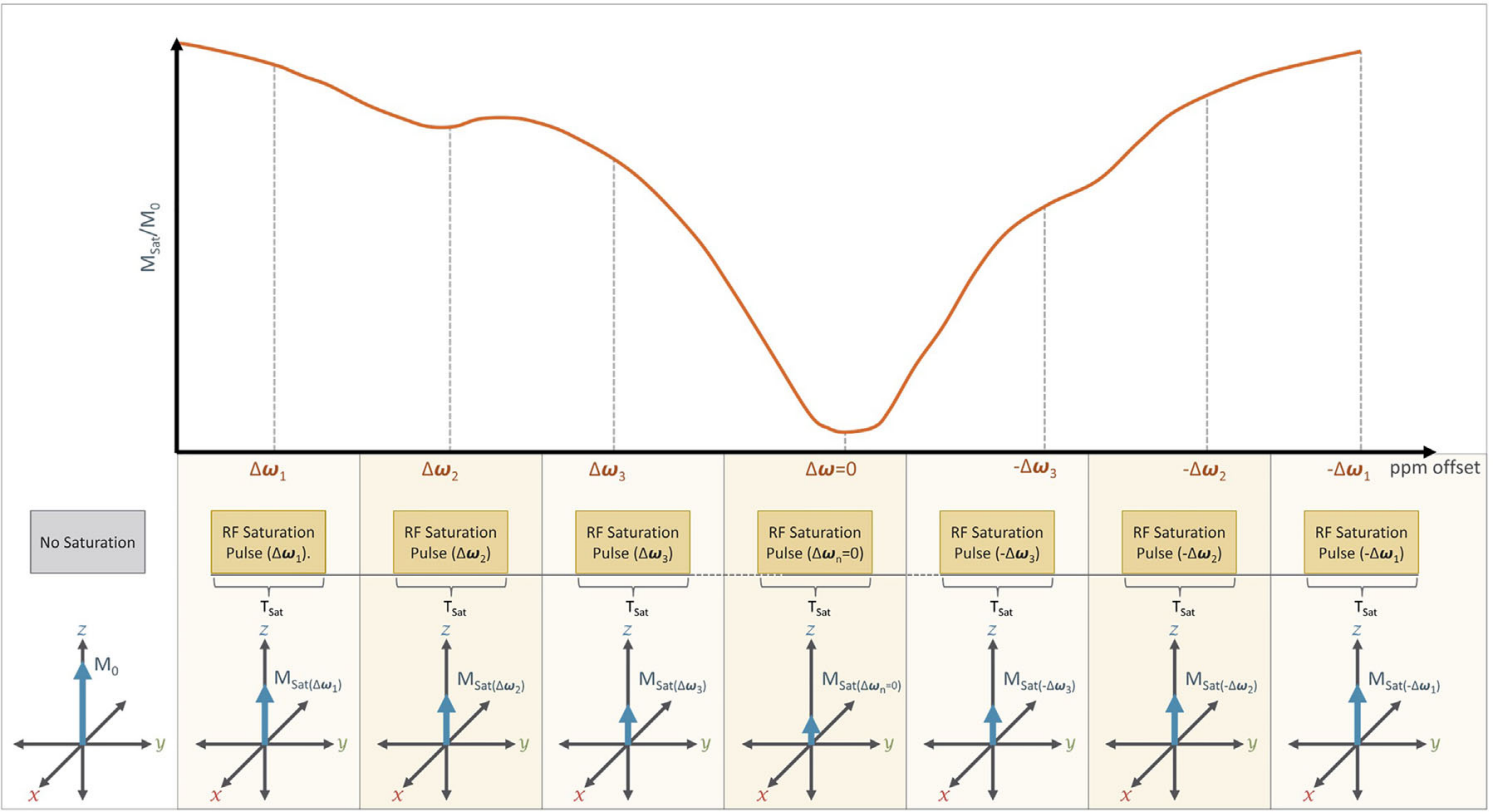
A typical CEST Imaging experiment: In practice, CEST imaging is performed using a series of RF saturation pulses that are applied at different offset frequencies (±Δ***ω***) which are measured relative to the Larmor frequency of free water (ω). Direct water saturation occurs when CEST is performed at the Larmor frequency of free water (i.e. Δ***ω***=0), which will result in a significant reduction in net magnetization (M_sat_) relative to the initial net magnetization (M_0_). The series of RF pulses are typically applied at and near the expected Larmor frequency of a desired solute proton (Δ***ω***_1_, Δ***ω***_2_, Δ***ω***_3_) and at frequency offsets (-Δ***ω***_1_,-Δ***ω***_2_, -Δ***ω***_3_) from water. The presence of a solute proton with a chemical shift (+Δ***ω***= Δ***ω***s) would therefore appear as a dip in the CEST spectrum at that chemical shift (+Δ***ω***s) relative to its opposite (-Δ***ω***s). For example, we can see such a dip in Δ***ω***_2_ relative to -Δ***ω***_2_.

**Figure 8:**
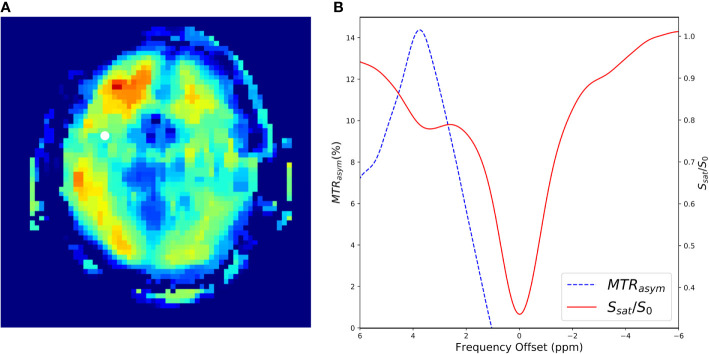
A CEST experiment collected in a human at 7T using an off-resonance RF pulse of B1 = 1.5 µT and a frequency sweep from −6 to 6 ppm with steps of 0.2 ppm. Data were collected using a 3D gradient echo sequence with a TE = 0.5 ms, TR = 3.8 ms. flip angle = 10, FOV = 22 cm × 22 cm × 15 cm, slice thickness=1.0 cm, matrix size = 64 × 64 × 10, NEX = 3.0. **(A)** Shows the resulting CEST_asym_ image generated with at Δ***ω*** at 3.5ppm. The white dot shows a region of interest where the CEST spectrum in **(B)** was generated. **(B)** Shows the CEST-spectrum plot in red and the CEST_asym_ spectrum in blue. The peak of the CEST_asym_ is at 3.5ppm corresponding to amide proton transfer (APT).

In many applications, even more RF saturation pulses are applied over a broad range of saturation frequencies (**Figure 7**) generating what is known as the Z-spectrum (3). An example CEST data set that targets the exchangeable protons of the amide group is shown in **Figure 8** along with the acquired Z-spectrum. This version of CEST imaging is referred to as amide proton transfer CEST (APTCEST) or simply APT and has been shown to be sensitive to pH. **Figure 8A** shows the APT CEST_asym_ map generated at 3.5 ppm corresponding to the amide proton chemical shift. The entire Z-spectrum (red line, **Figure 8B**) between ±6 ppm is shown on the right (**Figure 8B**) as well as the resulting CEST_asym_ curve (blue line, **Figure 8B**). This data set was collected on 7T MRI to exploit the increased spectral separation at the higher field strengths compared to lower field strengths. It should be noted that the CEST literature has adopted the convention of a chemical shift of 0 ppm corresponding to the water protons when displaying the Z-spectrum.

### Sensitivity and Specificity of CEST for Molecular and Metabolite Imaging

The CEST technique provides a novel way to detect endogenous compounds with exchangeable protons. These include several molecules that are known to reflect brain metabolism and neurotransmitters such as glutamate (4, 5), GABA (6), creatine (7), and lactate (8). The exchangeable protons on each of these compounds have a unique Larmor frequency. Hence, CEST experiments can be tuned to match the frequency of the desired molecular group. In the CEST literature, it is common to refer to the CEST experiments based on the probed molecule. For example, targeting glutamate with CEST is known as gluCEST while targeting creatine is known as CreCEST. **Table 1** lists several molecules that have been previously measured using CEST imaging as well as the factors proposed by the original manuscript authors thought to contribute to the findings observed. For example, studies have observed intracellular acidosis using APTCEST arising from anaerobic metabolism (9) resulting from stroke by comparing ipsilateral versus contralateral tissue.

**Table 1:** Varieties of CEST and their Uses.

Type of CEST Contrast	Previous Application (Citations)	Reported physiological changes
**APT CEST** pH weighted protein-peptide concentration Amide group @ 3.5 ppm	Ischemic penumbra in stroke (9–11)	Severe intracellular acidosis in ischemic core develops in part due to unopposed anaerobic ATP hydrolysis, with hypoperfusion and reduced bicarbonate buffering at acidic pH exacerbating the acidosis
	Tumor pH (mouse studies) reported that proportion of APT CEST signal originating from changes in protein concentration was approximately 66%, with the remaining 34% originating from changes in tumor pH. (12)	Tumor cells have reversed the pH gradient across the cell membrane with respect to normal cells, with a slightly alkaline intracellular pH (pHi) and an acidic extracellular pH (pHe). Tumors often have regions of acute and chronic hypoxia as a result of both an increased oxygen consumption rate of tumor cells compared with normal cells and hence altered pH.. Proteomic Analysis have revealed significant increase in the cytosolic protein concentration in the tumor, compared to normal brain regions.
	Tissue grading and classification in tumor (13–14)	The mean APT asymmetry ratio values highly correlated with tumor grades. Significant differences in APT asymmetry ratio were observed between tumor grades. Increased APT asymmetry associated with increased cell density, gliomas with microscopic necrosis.
	Alzheimer’s Disease vs healthy controls (15)	Elevated CEST asymmetry ratio in bilateral Hippocampus in Alzheimer’s may be due to increased cytosolic proteins and peptides (accumulation of amyloid plaques, neurofibrillary tangles, and neuronal loss).
	Parkinson’s Disease vs healthy controls (16)	Elevated CEST asymmetry in substantia nigra in Parkinson’s disease may be due to dopaminergic neuronal loss.
**GluCEST** Glutamate Amine group @ 3 ppm	Middle cerebral artery occlusion (MCAO) stroke model (17)	MCAO model lead to significant drop in pH resulting in elevated Glutamate concentrations due to increased proton exchange
	Tumor with BBB disruption and Glutamate injection (18)	Glutamate concentration in tumor cells increased due to glutamate injection
	Non-lesional Temporal Lobe Epilepsy (19)	Elevated Glutamate concentration correctly lateralized the temporal lobe seizure foci.
	Transgenic mouse models Alzheimer’s Disease vs wild type (20)	The excitatory neurotransmitter, Glutamate, is known to decrease in early stages of Alzheimer’s disease.
	Healthy controls vs Psychosis spectrum (4)	Abnormal glutamate neurotransmitter levels are implicated in progression of psychosis
	Differential gray:white (1.6:1 ratio) contrast in healthy brain (21)	Glutamate concentration map approximates the Glu receptor distribution reported in previous PET studies
	Mouse with MPTP model of Parkinson’s disease (22)	MPTP selectively kills the dopaminergic neurons in the substantia nigra pars compacta and striatum. Increased glial activity from astrocytes increase Glutamate concentration in Striatum after MPTP treatment
	Knock-in mouse model of Huntington’s disease vs wild type and heterozygous mice	Reduced Glutamate concentration in striatum in homozygous Huntington’s disease mice as a result of neuronal alterations.
**CrCEST** Creatine Amine group@ 1.8 ppm	Plantar flexion exercise within MRI scanner (7, 8)	Dynamic changes in creatine concentration in reposes to increased ATP consumption during exercise. Post exercise creatine recovery prolonged in mitochondrial disease group
	Post-exercise; Mitochondrial disease vs healthy controls	Creatine concentration in tumor cells varies from normal cells due to abnormal ATP metabolism in tumor
**MICEST** Myo-inositol Hydroxyl group @ 0.625 ppm	Transgenic mouse models of Alzheimer’s disease vs wild type control mice (23)	Elevated expression of activated glial cells from neuroinflammatory responses in Alzheimer’s disease pathology leads to increased myo-inositol concentrations
**GABACEST** GABA Amine group @ 2.5 ppm	Rat models of status epilepticus; pre-post epileptiform activity induced by kainic acid injection (24).	Epileptic seizure changes GABA concentrations
	Rat with brain tumor and blood-brain barrier disruption; pre-post GABA injection (25)	The disrupted blood-brain barrier in tumor region allowed to measure GABA concentration changes
**GlucoCEST** Glucose Hydroxyl group @ 0.6 to 1.5 ppm	pre-post injection of unlabeled glucose in Tumor (mice studies, human studies) (references below)	High rate of glucose uptake in tumors lead to glucose concentration changes
**Glyco CEST** Glycogen Hydroxyl group @ 0.75–1.25 ppm	Mouse liver studies with pre-post glucagon administration (26)	Glucagon stimulates glycogenolysis (glycogen to glucose conversion) and depletes glycogen
**LATEST** Lactate Hydroxyl group @ 0.4 ppm	Calf muscles pre-post exercise (15)	Dynamic lactate changes in exercising muscles
	tumor (27)	up-regulated lactate dehydrogenase (LDH) due to lactate metabolism in tumor

ppm, parts per million; APT, amide proton transfer; MPTP, 1-methyl-4-phenyl-1,2,3,6-tetrahydropyridine; ATP, adenosine triphosphate.

The specificity of CEST imaging for quantification of molecular concentrations has been extensively explored by many of the studies listed in **Table 1** using MRS measurements for validation. The CEST effect is known to be confounded by several undesirable factors. These include the contamination of the CEST_asym_ ratio by other saturation exchange mechanisms such as magnetization transfer (MT) and dipolar interactions such as nuclear Overhauser enhancement (NOE) (28, 29) and concentration from other metabolites whose chemical shift is close to the target metabolite. For example, imaging of glutamate is known to be confounded by GABA and Cr concentrations (17). Because of these confounding factors, the specificity of the CEST measurements are sometimes difficult to be interpret. Several spectral editing techniques are being developed to remove the confounding effects of these source of errors as discussed in *Other Confounding Effects*. Ignoring these confounding factors for the moment, the reliability of CEST measurements have been shown to have good scan/re-scan reliability (30–32).

### Strengths and Limitations of CEST Imaging

The key strength of CEST is that it can quantify several endogenous molecules with high resolution. This quantification can be performed using existing MR hardware and can readily be integrated into a multi-modal MRI study. Other MRI methods, particularly MRS, also provide the ability to study endogenous compounds. Relative to MRS, CEST has the advantage of measuring the endogenous molecules using the water signal, which is 5 orders of magnitude larger than the molecular concentrations directly observed with MRS. This allows CEST imaging to collect data faster and with higher spatial resolution than MRS. However, MRS has at least one significant advantage over CEST, which is its ability to quantify multiple compounds simultaneously, while CEST can typically only acquire one or two compounds at a time that must be defined *a priori*.

PET is another approach that is often used to study brain metabolism, but instead uses exogenous radioactive contrast agents. This provides PET with superior sensitivity and SNR as compared to CEST. In addition, PET imaging provides the ability to measure rates including blood perfusion, metabolic rate of glucose, and binding rate to a receptor. CEST in contrast is predominantly used to measure metabolite concentration. A detailed comparison of various CEST approaches and their closest PET alternatives was recently provided by Wu and colleagues (33). It should also be noted that PET and CEST can provide complimentary information. Therefore, the combined acquisition of these two metabolic imaging techniques is an exciting potential research direction facilitated by dual modality PET/MRI scanners, which are now commercially available.

Endogenous CEST imaging has the limitation that it can only be used to study metabolites with exchangeable protons whose exchange rate satisfy the condition that the exchange rate (k) is less than Δω_s_ (34). Moreover, the chemical shifts of these endogenous species are typically very small (Δω_s_ ≤ 10*ppm*), which make it prone to noise from undesirable sources. Some of these limitations can be addressed with the administration of exogenous contrast agents which have larger chemical shift (Δω_s_ ≈ 50–700 ppm). Such agents can be extremely useful for several unique applications such as metal ion detection, liposome labeling, nanoparticle/polymer labeling, RNA/DNA/protein-binding, temperature imaging, detecting enzyme activity, and identification of reporter genes. However, most of these agents have not been approved for use in humans and are currently limited to animal imaging studies. This review will not discuss the use of these CEST contrast agents and instead the reader is referred to Hancu et al. (35).

### Methodological Considerations for CEST

While CEST has great potential for studying certain molecules that are important to psychiatric research, there is substantial room for improvement in the acquisition and analysis of CEST data.

#### B_0_ Correction

CEST imaging can be sensitive to magnetic field inhomogeneities that shift the Larmor frequency and as a result the saturation pulses may be applied at the wrong frequency offset in some portions of the brain. One method of correcting for this magnetic field variation is to use the acquired Z-spectrum to identify the actual Larmor frequency of water independently for each voxel. This is done by identifying the RF saturation pulse, δω_s_, that has the strongest saturation of the water signal (instead of assuming that it is at 0 ppm). If the saturation of the water signal is sufficiently broad, a more rigorous approach may be required that involves using an additional scan where a finer sampling of the saturation pulses around the water signal is performed. This acquisition is then used to estimate the effective Larmor frequency on a voxel by voxel basis using a technique called water saturation shift referencing (WASSR) (36). The resulting estimates for S_0_ and ω_0_ are then used to correct the CEST_asym_ at each voxel.

#### Other Confounding Effects

One limitation of using CEST_asym_ to measure metabolite concentrations is that it introduces additional sources of errors due to the fact that macromolecular effects are asymmetric and lipophilic peaks exist on the right side of the water peak (i.e. -Δω_s_) that may confound the measurements (**Figure 7**). It has been shown that these sources of errors can dominate (37–39) the CEST_asym_ quantifications. Several techniques have been proposed to minimize these sources of error including double frequency irradiation (40, 41), and Lorentzian differences (29, 42). Lorentzian curve fitting has also been used by several groups to account for these potential sources of error (43–46). This technique fits a series of Lorentzian functions accounting for the CEST effect(s) of interest, nuclear Overhauser effect (NOE) or the dipolar interaction of protons with other nuclei species, magnetization transfer (MT), and bulk water to the Z-spectrum.

#### CEST Imaging Scan Time

A typical CEST imaging acquisition consists of several measurements performed at different Δ*ω*_s_ to sample the Z-spectrum and if acquired the WASSR data. The time to collect this data can be long and has often limited CEST measurements to a few slices. While a majority of the CEST imaging studies that have been reported in the literature have focused on limited coverage using 2D acquisitions, it is possible to perform whole brain acquisitions in approximately 10 min by combining fast scan methods such as echo-planar readouts and acceleration methods such as parallel imaging (28, 47). Image acquisition can also be accelerated using under-sampling techniques such as compressed sensing or machine-learning that minimize the number of measurements needed to reconstruct the data (28). Other improvements such as the ability to acquire data from multiple metabolites in a single session, regional specificity, improvements in SNR, and increased sensitivity would help provide a more comprehensive picture of brain metabolism and reduce scan times (48).

#### High Field Imaging

CEST studies can benefit from high field imaging. A higher magnetic field strength offers greater frequency separation (Δω_s_ measured in MHz) for water and metabolites. This improves quantification of the CEST effect by making it easier to avoid direct saturation effects and leakage from other metabolites with Larmor frequencies close to the target molecule. Since the exchange rate for CEST imaging and must satisfy the condition that the exchange rate must be less than Δω_s_ (34), CEST experiments at higher field strength scanners can target molecules with faster exchange rates than what is possible at lower fields. Moreover, with higher field strength, the T1 weighting increases making the CEST measurements more time efficient. Stronger magnetic fields also result in increased signal-to-noise-ratio which can be used to improve spatial resolution, increase anatomical coverage, or reduce image acquisition time.

#### Other Imaging Parameters

Like other MR imaging sequences, CEST imaging protocols require optimization of multiple imaging parameters including the number of RF saturation pulses, their step size, saturation pulse duration, repetition time, and flip angle. These parameters need to be tuned for each metabolite and field strength. The specifics of this are outside the scope of this review, however a number of review articles on CEST imaging already exist, which include discussions of parameter optimizations (34, 49–52). Finally, optimal approaches for quantification and challenges across field strengths are also not discussed here in the interest of space but have been previously reviewed in Kim et al. (52) and Wu et al. (33).

## Acquisition and Analysis Of T1ρ

The phenomenon of proton exchange is also exploited in T1ρ imaging. In contrast to CEST, which uses a saturation RF pulse to tune to the Larmor frequency of the exchangeable solute protons, T1ρ relies on generating a secondary magnetic field to become sensitive to proton exchange mechanisms. This sensitization is achieved using an RF pulse known as a “spin-lock” pulse, which generates a secondary weak magnetic field B_1_ perpendicular to the main magnetic field B_0_. The spin-lock (SL) RF pulse is applied at the Larmor frequency of the water protons and “locks” the net magnetization in the transverse plane, causing the magnetization to precess around the new B_1_ field. The frequency of precession, ω_sl_, about the spin-lock B_1_ magnetic field is given by

(7) $$\omega_{sl}=\gamma B_{1}$$

which is obtained by replacing the scanner magnetic field strength, B_0_, with the spin-lock field strength, B_1_. This new precession frequency about B_1_ is typically in the range of 100 Hz to 3 kHz and is much slower than that around the main magnetic field (typically in the range of MHz). The slower precession allows the magnetization to be sensitive to proton exchange processes that also occur in this range. Consequently, the relaxation of the magnetization under the influence of B_1_ is made sensitive to proton exchange.

**Figure 9:**
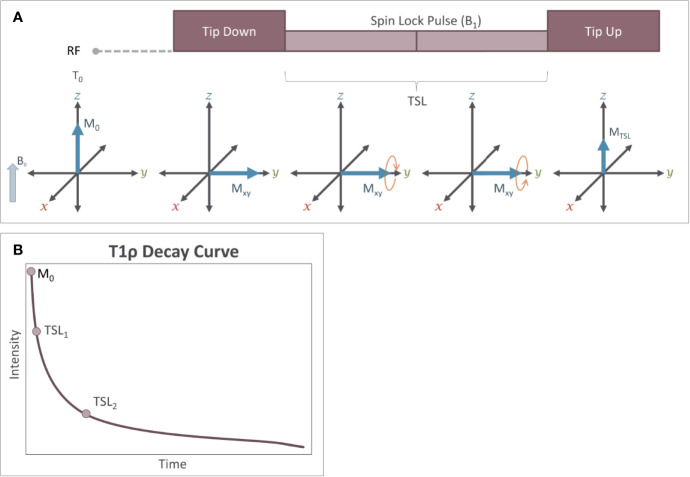
Measurement of T1ρ relaxation is performed using a magnetization preparation block **(A)** consisting of a series of RF pulses. The first RF pulse tips the magnetization 90˚ from the Z axis into the transverse plane. The second RF pulse is the spin-lock pulse (SL) which generates a small magnetic field (B_1_) in the rotating (transverse) plane. The spin-lock pulse is often broken into two equal duration RF pulses that are 180˚ out of phase. The third RF pulse is a −90° pulse, which restores the magnetization to the Z axis. During this spin-lock pulse, the net magnetization of the protons precesses around the B_1_ field. Because the strength of B_1_ is relatively small, this precession occurs at a frequency ω_SL_, which is substantially slower than precession around B_0_ and that is similar to the frequency of several slow processes including proton exchange. Because of this, over time, a loss of magnetization occurs in the transverse plane. **(B)** Measuring the loss of signal resulting from T1ρ relaxation requires that at least two different spin lock durations (TSL) are applied during the experiment (i.e. the steps in A are repeated). T1ρ follows an exponential decay curve and is calculated by using these different TSL values to fit the exponential decay function: M_TSL_ = M_0_e^-TSL/T1ρ^.

The basic T1ρ imaging scan is implemented using a block of three RF pulses as shown in **Figure 9A**. The first 90-degree RF pulse tips the longitudinal magnetization into the transverse plane. The second RF pulse, (the spin-lock pulse), is applied in the transverse plane at low amplitude. While the spin-lock (SL) pulse is being applied, the B_1_ field itself rotates around the axis of B_0_ at the Larmor frequency defined by the main magnetic field. Thus, it is easier to understand the influence of the spin-lock pulse on the magnetization by considering this process in the “rotating frame” from which the imaging technique gets its name. This can also be conceptualized as the perspective from the proton as it precesses around the B_0_ field and is similar to a human observing events while standing on earth as it precesses around the sun. In the rotating frame, the B_1_ field appears to be stationary (despite the fact that in absolute terms, it is rotating along the transverse plane around the axis of B_0_), and the net magnetization precesses around the B_1_ field. The SL pulse is typically delivered in two segments, with the second segment being delivered at the opposite phase from the first segment. Finally, the third RF pulse returns the magnetization to the longitudinal axis using a −90-degree RF pulse. The T1ρ preparation block is then followed by a magnetic field gradient that is applied to destroy residual transverse magnetization before using a standard imaging scheme to measure the magnetization stored in the longitudinal axis.

During the spin lock pulse, some relaxation of the magnetization occurs due to proton exchange. This relaxation produces an exponential decay in the signal intensity and is where the technique gets its name (T1 relaxation in the rotating frame). The relaxation time constant, T1ρ, can be calculated by measuring the change in signal intensity that occurs when applying at least two different spin-lock durations and fitting the data to an exponential decay curve (equation 8, **Figure 9B**)

(8) $$S(TSL)=S_{0}e^{-TSL/T1\rho }$$

**Figure 10:**
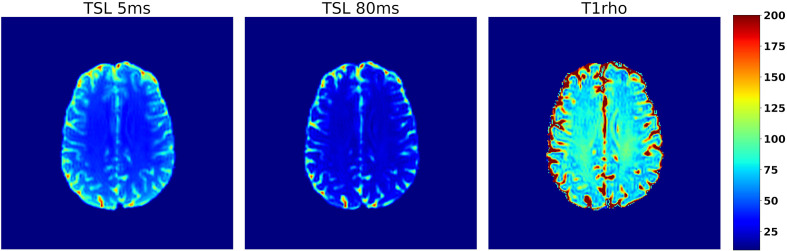
Axial slice from a T1ρ data set collected from a human participant using a 3D spin-echo sequence with a spin-lock amplitude of 14.1uT. Images A and B show the same axial slice collected using spin-lock durations of 10 and 80 ms respectively, which illustrates the decay due to T1ρ relaxation that occurs while applying the spin-lock pulse. The resulting T1ρ map is shown in C) and the color scale on the far right corresponds to the relaxation times in C.

In Equation 8, S_0_ is the signal with no spin-lock pulse applied; TSL is the time duration of the spin-lock pulse. While the quantification of T1ρ requires a minimum of two measurements with different TSL values (**Figure 10**), acquiring data from more than two TSL values increases the accuracy of the T1ρ quantification (53). Since the spin-lock pulse slows the relaxation process in the transverse plane, the T1ρ relaxation time is longer than the T2 relaxation time for a given sample. As the amplitude of the spin-lock pulse (B_1_) approaches zero, T1ρ relaxation times will get shorter and will approach T2 relaxation times. Similarly, as the amplitude of the spin-lock pulse increases the T1ρ relaxation times will increase.

### Sensitivity and Specificity of T1ρ for Metabolic and Molecular Imaging

T1ρ imaging can be used to measure low-frequency biological processes that occur on the same time frequency as the applied spin-lock pulse that is not feasible using conventional T1 or T2 imaging. Since the frequency of precession resulting from the spin-lock pulse is typically in the range of 100 Hz to 3 kHz, T1ρ imaging is sensitive to exchange processes that fall in this range. These include chemical exchange as well as spin-spin coupling, dipole-dipole interactions, and diffusion (54). Importantly, the frequency of precession can be “tuned” to favor a particular process. This is analogous to how diffusion gradients are used in diffusion weighted MR imaging where the sensitivity to water motion is dependent on the amplitude of the gradients applied.

**Figure 11:**
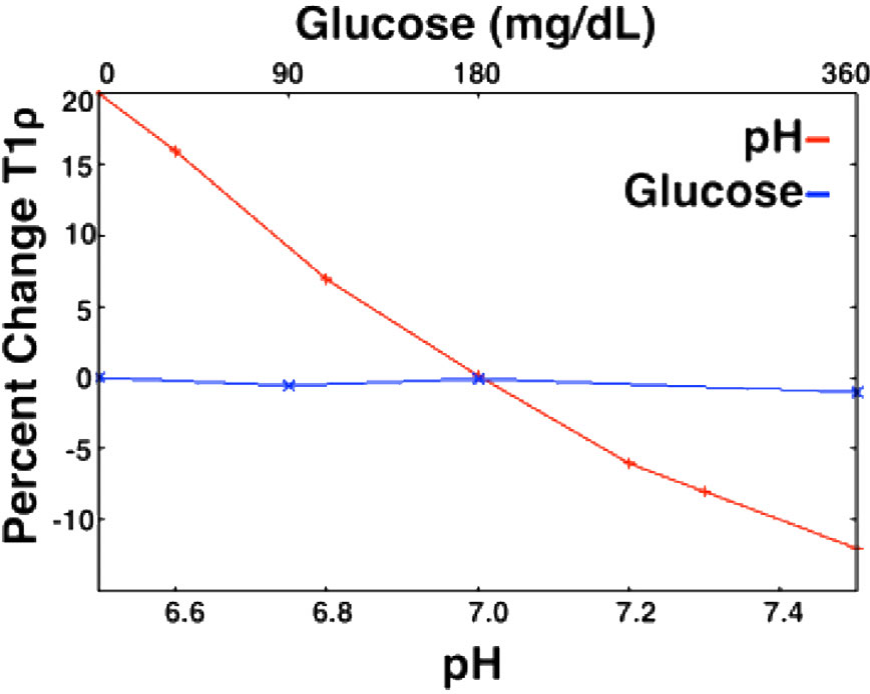
Relationship between T1ρ and metabolites (pH and glucose) in an egg white albumin phantom. T1ρ shows a much greater response to pH changes in the physiological range as compared to glucose in physiological range (59).

Notably, T1ρ may be sensitive to a broader range of exchange processes than CEST, since it is not tuned to a particular group of exchangeable protons. Proton exchange with amide, hydroxyl, and amine groups are all thought to contribute to T1ρ relaxation (55). Thus, the T1ρ relaxation time is affected by changing the concentrations of these solutes and the rate at which they can exchange with water protons. T1ρ values has been shown to be sensitive to the concentrations of molecules including glucose (56–58) in phantom studies. T1ρ imaging is also sensitive to pH (**Figure 11**) (56, 59, 60), the amount of water that is present in tissue (56, 61), macromolecular density (60), and temperature (59). Several studies have assessed the reliability of T1ρ relaxation times at 3T and found good scan/re-scan reliability with differences on the order of 2% or less (59, 62) between scans.

**Table 2** lists previous studies that have used T1ρ imaging in the context of brain imaging. Most of these studies have been in human subjects and observed T1ρ relaxation time differences when comparing various psychiatric and neurological disorders to control subjects. This has included increased T1ρ relaxation times observed in subjects with mild cognitive impairment and Alzheimer’s Disease as compared to controls that the authors attributed to differences in macromolecular content, oxidative stress, and pH secondary to the disease pathophysiology (62, 67–71). In Parkinson’s disease T1ρ relaxation times in the substantia nigra were increased compared to control subjects likely due to tissue degeneration (63). In multiple sclerosis three studies have all reported increased T1ρ relaxation times in cerebral white matter likely reflecting the associated de-myelination (64, 65, 78). And in pre-manifest Huntington’s disease (HD), T1ρ relaxation times in the striatum were increased compared to controls and correlated with proximity to predicted symptom onset, likely reflecting degeneration-induced changes in pH and/or glucose (79).

**Table 2:** Use of T1ρ in Neurology and Psychiatry Research.

Title	Sample	Summary Findings
**Parkinson’s Disease**		
**T_1ρ_ and T_2ρ_ MRI in the evaluation of Parkinson’s disease** (63)	9 moderate PD, 10 HC	Increased T1ρ in substantia nigra of PD participants. Motor asymmetry was strongly correlated with asymmetry in T1ρ.
**Multiple Sclerosis**		
**Magnetization transfer and adiabatic T1ρ MRI reveal abnormalities in normal appearing white matter of subjects with multiple sclerosis** (64)	9 Relapsing - Remitting MS, 7 HC	Adiabatic T1ρ increased ~6% in normal appearing white matter in MS participants vs. HC.
**In vivo quantitative whole-brain T_1_ rho MRI of multiple sclerosis** (65)	10 Clinically Isolated MS, 13 Relapsing-remitting MS, 24 age-matched HC	T1ρ contrast in WM were elevated compared with controls. WM lesion T1ρ correlated with disease duration and provided better contrast than T2
**Alzheimer’s Disease**		
**In Vivo Measurement of Plaque Burden in a Mouse Model of Alzheimer’s Disease** (66)	6 APP/PS1 transgenic and 6 wild-type mice	T1ρ relaxation decreased in 12 and 18-month-old transgenic mice of AD model, compared to age-matched controls Changes in macromolecular content from increased Aβ deposition, decreased blood flow/volume, associated with AD
**Early Marker for Alzheimer’s Disease: Hippocampus T1rho (T_1ρ_) Estimation** (67)	49 AD, 48 MCI, 31 HC	T1ρ increased in hippocampus in AD, MCI compared with healthy controls
**T1_ρ_ MRI of Alzheimer’s Disease** (68)	14 AD, 11 MCI and 16 HC	T1ρ increase in both the GM and WM in the MTL in AD patients over age-matched controls.
**T_1ρ_ MRI in Alzheimer’s Disease: Detection of Pathological Changes in Medial Temporal Lobe** (69)	48 AD, 45 MCI, 41 HC	T1ρ increased in medial temporal lobe in AD, MCI compared with healthy controls
**T1rho (T_1ρ_) MR imaging in Alzheimer’ disease and Parkinson’s disease with and without dementia** (70)	53 AD, 62 PD, 11 PD with Dementia, 46 HC	T1ρ increased in AD, decreased in PD. AD may disrupt molecular interactions between bulk water and slowly tumbling macromolecules in the extracellular space
**T1rho MRI and CSF biomarkers in diagnosis of Alzheimer’s disease** (71)	27 AD, 17 MCI, 17 HC	T1ρ was nearly as effective as CSF biomarkers at predicting AD and MCI.
**Epilepsy/Seizure**		
**Progression of Brain Damage after Status Epilepticus and Its Association with Epileptogenesis: A Quantitative MRI Study in a Rat Model of Temporal Lobe Epilepsy** (72)	20 adult male Harlan Sprague–Dawley rats	T1ρ and T2 in connected brain regions were increased acutely and several weeks after amygdala-stimulation induced seizures. MRI did not predict severity of seizures
**MRI Biomarkers for Post-Traumatic Epileptogenesis** (73)	34 injured and 16 control adult male Spague-Dawley rats	T1ρ better than T2, diffusion at predicting seizure susceptibility following (9 days and 23 days) induced TBI. Diffusion was the best predictor after 2 months.
**Panic Disorder**		
**Functional T1ρ Imaging in Panic Disorder** (74)	13 PD, 13 HC	Increased T1ρ in visual cortex and anterior cingulate in response to flashing checkerboard task in panic disorder
**Bipolar Disorder**		
**Brain Abnormalities in Bipolar Disorder Detected by Quantitative T1ρ Mapping** (75)	15 BD I, 25 HC	Quantitative T1ρ increased in cerebral white matter and cerebellum in BD. There was no difference between BD participants being treated with lithium and HC.
**Alterations of the cerebellum and basal ganglia in bipolar disorder mood states detected by quantitative T1ρ mapping** (76)	40 BD I, 29 HC	Quantitative T1ρ increased in BD compared with HC. Depressed and Manic groups had decreased T1ρ in basal ganglia when compared with healthy or Euthymic groups.
**Relationship altered between functional T1ρ and BOLD signals in bipolar disorder** (77)	39 BD I, 32 HC	Functional T1ρ and BOLD were strongly related during flashing checkerboard task, but this relationship was weaker in BD

PD, Parkinson’s disease; HC, healthy control; BD, bipolar disorder; AD, Alzheimer’s disease; MCI,-mild cognitive impairment; MS, multiple sclerosis; CSF, cerebrospinal fluid; TBI, traumatic brain injury; WM, white matter

### Strengths and Weaknesses of T1ρ Imaging

T1ρ imaging shares many of the same strengths as CEST; namely that it can be performed using typical MRI hardware, does not require injected contrast agents, and the SNR of the technique relies on the signal from water which exists in high concentrations. However, T1ρ can also be acquired with a higher spatial resolution and more quickly than CEST since a relatively small number of measurements are needed. Quantitative whole brain T1ρ measurements can be obtained in 6 min or less with a 2-mm isotropic resolution. Another strength of T1ρ imaging is the relative short preparation block on the order of 100 ms as compared to CEST imaging where the long saturation pulses can often last a second or more. As a result, the T1ρ preparation block can be added to fast imaging techniques such as echo-planar imaging and used to assess functional brain activity (80, 81). Studies of functional T1ρ have shown that the signal peaks more quickly following a stimulus (82) and that the T1ρ signal is more spatially constrained than the blood-oxygen-level-dependent (BOLD) contrast that is typically used for functional imaging (56, 83). These characteristics hold the potential to increase the temporal and spatial resolution of functional imaging. Furthermore, the T1ρ signal reaches a plateau during stimulation rather than dropping off and lacks a post-stimulus undershoot, both of which are present in BOLD response (56). Furthermore, it is possible that the source of functional T1ρ is more directly linked to neuronal activity than the BOLD signal which relies on a hemodynamic response (77, 84). Because T1ρ is highly sensitive to pH in the physiological range, one possibility is that the functional T1ρ signal reflects local pH dynamics (71). Alternative possibilities include changes in cerebral blood volume (CBV), which accompany the functional hemodynamic response, and may also contribute to the functional T1ρ signal (82, 83). However, two studies that suppressed the blood signal using contrast agents (60) and saturation pulses (80) found that suppressing the blood signal reduced only a portion of the functional T1ρ signal leaving most of the signal unchanged. Thus, CBV likely underlies some of the functional T1ρ signal, but approximately 2/3 of the signal seems to come from other sources that may include glutamine, glucose, and pH (56, 82, 83).

A weakness of T1ρ is likely its lack of specificity. Given the potential broad sensitivity of T1ρ imaging to multiple metabolites as well as pH, it may be best to currently consider T1ρ as an overall marker for metabolic activity and not a quantification of any single metabolite. Hence, its utility in determining changes driven by a single molecule must be performed in a highly controlled experimental condition. While, it has been shown that collecting multiple spin-lock amplitudes as well spin-lock durations can help to identify individual metabolic contributions (54), more work is still needed to identifying how different imaging parameters effect the sensitivity of T1ρ to different mechanisms or molecules especially *in vivo*. The factors contributing to T1ρ relaxation time is a highly relevant topic that is still being investigated by several groups (54, 59, 60).

T1ρ imaging should be considered complementary to PET and MRS techniques. PET imaging is specific regarding the metabolic processes or receptors that it is targeting based on the radiotracer administered. Similarly, MRS can quantify several brain metabolites that contain hydrogen or phosphorus atoms with well-known chemical shifts near the Larmor frequency of the particular metabolite. T1ρ is not specific and instead is influenced by factors that may influence proton exchange. However, T1ρ is a low-cost technique to screen for potential metabolic changes that could then be followed up with either PET or MRS. For example, finding a T1ρ difference in the cerebellum could be followed up with targeted single voxel or multi-voxel ^1^H and ^31^P measurements in that region to help understand the underlying metabolic differences. Likewise, PET imaging could be used to probe differences in glucose metabolism. With the commercial availability of combined MR/PET scanners, simultaneous T1ρ and PET studies are now possible and could provide new insights into the biological processes that drive the T1ρ differences that have been observed to date.

### Methodological Considerations for T1ρ Imaging

#### Magnetic and RF Field Inhomogeneities

T1ρ imaging is susceptible to B_1_ and B_0_ field inhomogeneities. B_1_ inhomogeneity leads to a deviation of the expected flip angles and result in banding artifacts in the acquired images. Common approaches for avoiding this include a rotary approach where the phase of the second half of the spin-lock pulse is reversed. However, this approach makes the imaging sensitive to relaxation in the plane perpendicular to the spin-locking RF pulse, which needs to be accounted for using multi-exponential decay models. Phase cycling methods (85) and adiabatic RF (86, 87) pulses have also been proposed to compensate for the B_1_ inhomogeneities. B_0_ compensation can be achieved by using a high-amplitude spin-lock pulse. However, this results in tissue heating and elevated specific absorption rate (SAR) issues. A solution that overcomes both B_1_ and B_0_ inhomogeneity is to insert a 180° RF pulse in the middle of the spin-lock pulse to compensate for off-resonant effects (88). Some residual B_1_ effects may still exist and more complex spin-lock preparation blocks have been proposed (85).

#### T1ρ Imaging Time

Multiple images with different spin‐lock times (TSLs) are needed to quantify T1ρ relaxation times, and the preparatory nonselective spin‐lock pulse precludes use of many rapid multi‐echo and multi‐slice strategies due to heating and saturation constraints. Several techniques have been investigated to reduce acquisition times, including rapid pulse sequences (89), parallel imaging (75, 90), keyhole imaging (91), and reduction of the number of TSLs required for specific applications (53). Johnson et al. proposed an optimal sampling of the spin-lock durations when the approximate values of the T1ρ relaxation times are known in advance (92) to improve the T1ρ imaging efficiency. Owusu et al. have also recently shown that a spin-lock amplitude of 14.1µT (600Hz) may be optimal for *in vivo* imaging the brain at 3T when considering tissue heating / SAR constraints (59).

#### T1ρ in Functional MR Imaging

There are several ways in which functional T1ρ imaging could be improved. For example, it remains unknown whether there is a stereotypical response function for functional T1ρ that would serve a similar purpose as the hemodynamic response function does for BOLD imaging. Such a function could be used to improve data collection, allow for more complicated task designs, and would improve statistical analysis of functional T1ρ data. T1ρ imaging sequences are also currently less developed, which limits spatial coverage as compared to BOLD imaging due to time needed for the spin-lock pulses in the imaging sequence. However future innovations in sequence development such as the integration of simultaneous multi-slice (SMS) acquisition may help to overcome this limitation.

#### High Field Imaging

High field imaging can be used to improve the SNR for T1ρ studies. Furthermore, detection of exchange from certain molecules such as glutamine and glucose (56) may require ultra-high magnetic fields and large spin-lock amplitudes (e.g. 9.4T; SL amplitudes: 125–4,000 Hz) in order to achieve sufficient signal or to appropriately match their exchange rates, which may be difficult to achieve *in vivo* due to subject heating / SAR constraints.

## Prior Uses of Cest and T1ρ To Study Psychiatric Disorders

Proton-exchange imaging has been used in the study of neurological disorders. CEST and T1ρ have detected pathophysiological changes in stroke, multiple sclerosis, epilepsy, Alzheimer’s disease, Huntington’s disease, and Parkinson’s disease. These studies are listed in **Tables 1**, **2** to illustrate the broad utility for proton exchange imaging in neuroscience. In addition, because many of the compounds detectable by CEST and T1ρ have been suggested to be abnormal in psychiatric illnesses, there is also great potential for CEST and T1ρ imaging in psychiatric research. Below we summarize how proton exchange techniques have been used thus far in psychiatric research and highlight potential future opportunities for development of proton exchange techniques that would enhance their use in the field.

### CEST

^1^H-MRS studies have suggested that glutamate and glutamine levels in the brain may be abnormal in patients suffering from schizophrenia; both increases and decreases have been reported (93, 94). Recent studies have thus tested whether CEST might also detect differences in glutamate levels in schizophrenia. One study used CEST to measure glutamate in research participants with psychosis, including 5 with schizophrenia, 14 at familial high risk for developing psychosis, and 17 healthy controls (4). Lower glutamate concentrations were detected in both the schizophrenia and high-risk participants in both cortical and subcortical brain regions. Moreover, the severity of clinical symptoms correlated with the low glutamate concentrations. A second study used CEST to test glutamate concentrations in the olfactory cortex, and found glutamate was increased in participants with schizophrenia compared to a control group (5). Because sample sizes in these studies were so small, their results remain inconclusive; however, they highlight the potential utility of CEST imaging in psychiatry research.

### T1ρ

**Figure 12:**
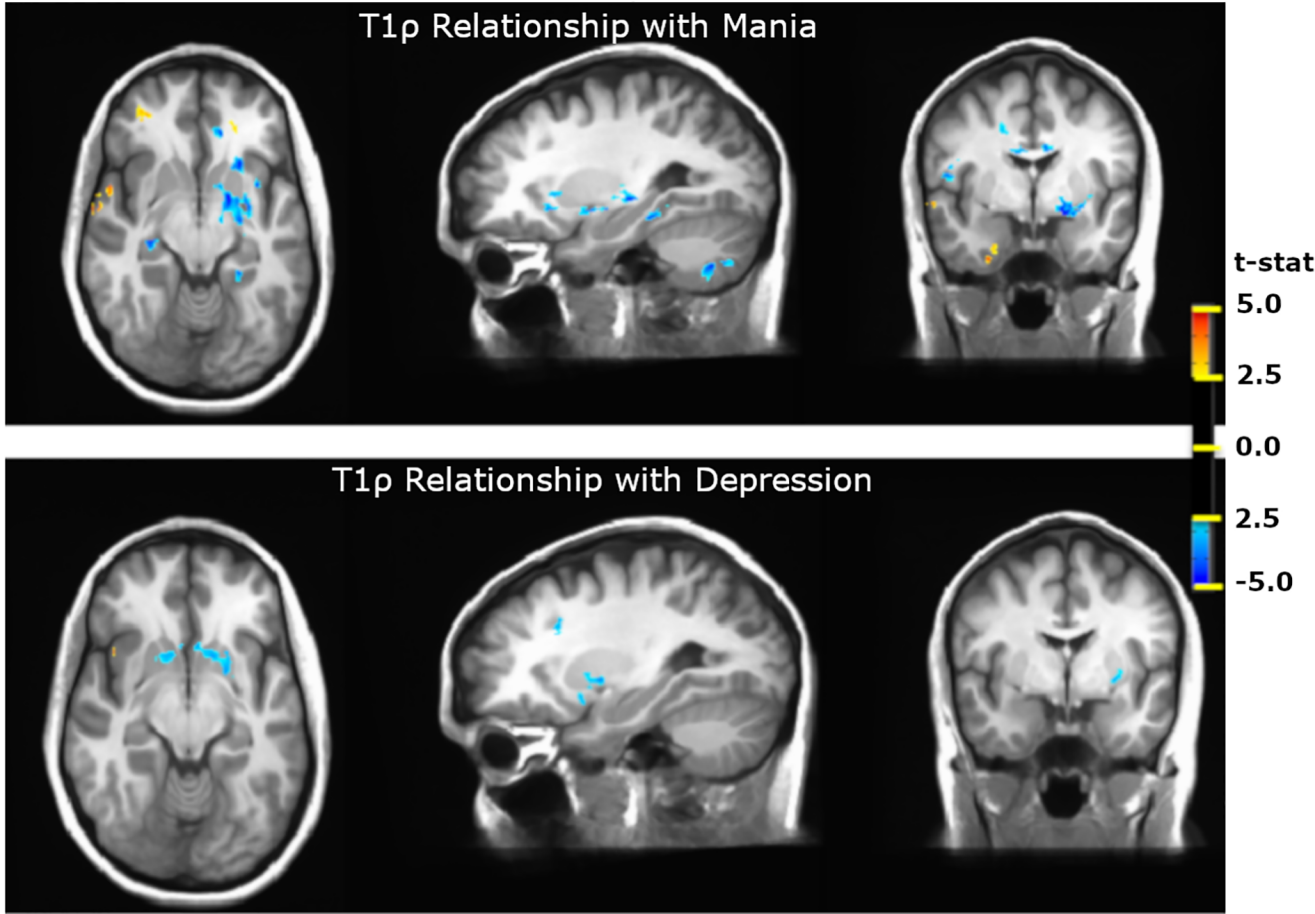
T1ρ differences in Bipolar Disorder and Bipolar Disorder Mood States. T1ρ is decreased in the basal ganglia and thalamus in both manic and depressed mood states. During mania, T1ρ is also reduced in the hippocampus and cerebellum and is increased in inferior frontal and temporal cortex (76).

A few studies have tried T1ρ to probe underpinnings of psychiatric illnesses. For example, Johnson et al. used whole-brain quantitative T1ρ imaging to study participants with bipolar disorder in the euthymic state compared to healthy control participants (75, 95). Interestingly, T1ρ relaxation times were elevated in participants with bipolar disorder, specifically in cerebral cortex (white matter) and cerebellum (white and gray matter). The white matter abnormalities were identified in regions that had not been previously implicated in bipolar disorder by other imaging modalities, including corpus callosum, sagittal striatum, superior longitudinal fasciculus, and cerebellar peduncle. Brain volumes in these regions were normal, suggesting that cellular loss was not the source of the T1ρ abnormalities. A comparison with inflammatory markers further suggested that inflammation was not likely the source of the increased T1ρ signal. One potential cause might be altered metabolism, as previous studies in bipolar disorder using MRS-based techniques have found decreased pH, increased glucose concentrations, and evidence of altered cellular metabolism (96–99). Interestingly, Johnson et al. found that the elevated T1ρ signal in the cerebellum in bipolar disorder was absent in participants taking lithium, suggesting the intriguing possibility that lithium may correct the pathophysiology underlying the abnormal T1ρ signal (75, 95) . Finally, Johnson et al. have also observed differences in T1ρ relaxation times that were associated with mood (76). When comparing subjects with bipolar disorder in depressed and manic mood states to those in a euthymic mood state, the subjects in depressed and manic mood states had shorter T1ρ relaxation times in the basal ganglia and thalamus (**Figure 12**). These different patterns of T1ρ signal with different mood states suggest that T1ρ may be useful for pinpointing specific changes in brain function and/or metabolism during specific mood states. Further study using a longitudinal design would help to better understand these changes.

### Functional T1ρ

Functional T1ρ imaging has been explored recently as well (74, 77, 84). In a study by Magnotta et al., participants with panic disorder and healthy controls underwent both functional T1ρ and BOLD imaging during a flashing checkerboard task (74). T1ρ responses were significantly increased in the visual cortex and significantly decreased in the anterior cingulate cortex in participants with panic disorder compared to healthy controls. Increased T1ρ responses correlated with panic symptom severity quantified using the Beck Anxiety Inventory. Interestingly these differences were not detected in the BOLD data, suggesting that activity-evoked T1ρ responses reflect mechanisms distinct from the hemodynamic response. Consequently, activity-evoked T1ρ responses may help identify abnormalities in brain function that BOLD cannot. Consistent with this possibility, a recent study investigated the relationship between the activity-evoked T1ρ response and the BOLD signal in research participants with bipolar disorder (77). Interestingly, the functional T1ρ -to-BOLD relationship was weaker in people with bipolar disorder compared with healthy controls (77), suggesting that the mechanisms underlying the functional T1ρ and BOLD responses are different and somehow differentially affected by the illness. These observations suggest that for some applications functional T1ρ may be better than BOLD. Moreover, combining the two methods may provide a more thorough functional assessment of brain activity.

## Potential Uses for Cest and T1ρ in Psychiatric Imaging

Given the strengths of proton exchange imaging techniques, they may serve as powerful tools for imaging important aspects of psychiatric illness. Outlined below are a few of the areas where proton exchange imaging could be applied to studies of psychiatric disorders and potential new insights that could be gained.

### Acidosis (or pH Sensitive Neuroimaging)

Abnormally acidic pH in brain tissue can result from a variety of complex physiological and pathophysiological processes affecting proton generation and/or pH buffering (100–102). These processes include respiration, blood flow, metabolism, and inflammation. Thus, finding pH abnormalities in the brain could be an important step toward understanding an illness and might suggest a variety of potential causes. Because a discussion of the many pathophysiological causes of abnormal pH is too broad for this review, we instead point to specific examples of psychiatric illnesses for which altered brain pH has been implicated. These include panic disorder (103–106), bipolar disorder (98, 107, 108), and schizophrenia (98, 107). MR spectroscopy-based studies have confirmed pH changes and lactate changes in schizophrenia and bipolar disorder (107). Abnormal pH buffering and metabolism have been suggested to cause the acidosis in panic disorder (74, 104, 105). Altered pH or pH dynamics have the potential to alter physiology and behavior through pH sensitive receptors and channels (100) or by directly affecting macromolecules (109). The ability to detect these pH differences in the functioning brain may be key to gaining insight into physiology and pathophysiology of psychiatric disorders. Moreover, localized changes in pH might serve as a valuable biomarker. CEST imaging is sensitive to pH since the exchange rates are influenced by pH. Taking advantage of this sensitivity, Zhou et al. demonstrated the utility of APTCEST for pH sensitive imaging studies (110). Several studies have since then employed APTCEST to detect pH reduction in acute ischemic acidosis (111, 112). While pH sensitivity of proton exchange is detectable via other flavors of CEST including GluCEST and LATEST, APTCEST remains the most widely employed pH-sensitive CEST technique because it can be readily performed at 3T. Absolute quantification of pH is not currently possible *in vivo* without the use of a pH reporting CEST contrast agent (113).

T1ρ imaging is also sensitive to pH. Systemic manipulation of pH by using CO_2_ inhalation caused increase in T1ρ relaxation times (83). Similarly, the T1ρ has shown to increase in human brain in response to visual flash checkerboard (82), a task that has been shown to decrease pH detected by ^31^P spectroscopy and to increase lactate/creatine ratio by ^1^H MR Spectroscopy (96).

### Neurotransmitters

Glutamate and GABA are the most common excitatory and inhibitory neurotransmitters in the brain and are thought to have a role in several psychiatric disorders. Postmortem and MRS studies have reported altered glutamate levels in diverse brain areas in individuals with mood disorders (114, 115) and glutamatergic abnormalities are also thought to have a role in schizophrenia (116). Several novel classes of antidepressants and mood stabilizers target glutamatergic activity (117). However, it is difficult to study glutamate concentrations either dynamically or throughout the brain using MRS. GluCEST may provide an alternative method for measuring differences in glutamatergic function and to assess relationship between glutamate levels and clinical symptoms or in response to therapies.

Similarly, GABA has been implicated in schizophrenia, bipolar disorder and major depression through several postmortem (118, 119) and MRS studies (120–122). GABA plays an important role in inhibition in the brain and is thought to regulate functions that have been disrupted in psychiatric illness including oscillatory rhythms (123), information processing (124), and sensory gating (125). GABACEST may therefore be a useful tool in measuring the activity of GABAergic neurons in psychiatric illness.

### Neuroinflammation

MICEST is sensitive to myoinositol, a glial cell marker that can be used as an indicator for neuroinflammatory response. Neuroinflammatory microglia activity has been reported in autism (126, 127), mood disorders (128), and schizophrenia (129). Gene expression studies in *postmortem* brain tissue have also indicated glial abnormalities, including reduced expression of astrocyte related genes in the cerebral cortex of individuals with major depression (128) and oligodendrocyte related transcripts in bipolar disorder (130). *Postmortem* studies have also observed reduced glial number in the hippocampus (131) and dorsolateral prefrontal cortex in alcoholism, with and without comorbid mood disorder (132). Therefore, MICEST may be a useful neuroimaging tool for the study of glial cells *in vivo*, particularly their development and response to treatments.

#### Metabolism

Creatine (Cr) is an important molecule for energy homeostasis and metabolism and altered creatine levels have been reported in schizophrenia, bipolar disorder, and other mood disorders (133, 134). While creatine is difficult to distinguish from phosphocreatine (PCr) using ^1^H MRS, phantom studies and muscle energetics studies using pre and post-exercise have shown that the contribution of PCr is minimal in the signal measured by CrCEST (7, 8), allowing for more accurate measurement of Cr. Similarly, the direct measurement of glucose (GlucoCEST), lactate (LATEST) and glycogen (Glycogen CEST) can also be used to accurately and quickly measure brain metabolism. GlucoCEST could also be used in conjunction with exogenous 3-O-methyl-D glucose, a method that has been shown to be useful for identifying tumors in rodent models (135, 136) and in human glioma patients (18), but that could also be used to measure the uptake of glucose in psychiatric disorders. 3-O-methyl-D-glucose is non-radioactive and biodegradable, and the dynamic imaging is shown to provide comparable results to that of FDG PET studies (18, 135, 136). These approaches are currently limited to ultra-high field strengths (18).

### Multimodal Imaging

Because CEST and T1ρ can be performed in the same imaging session as other common MRI techniques, it may be useful to use it as part of a multi-modal approach. For instance, combining T1ρ imaging with T1 and T2-weighted structural images may provide insight into the neurobiological underpinnings for the anatomical differences that have been identified in a number of psychiatric disorders. Likewise, the use of quantitative T1ρ may improve the interpretability of DWI data and may be used to help clarify whether differences in tissue diffusivity are related to altered tissue integrity (i.e. fewer/smaller cells), inflammation (more fluid), or tissue organization. Perhaps most interestingly, the use of functional T1ρ imaging in conjunction with BOLD imaging may allow researchers to separate metabolic and hemodynamic components of the functional response to task stimuli (77).

Ultimately, further work is needed to identify when to use T1ρ or CEST imaging most effectively. Currently, for example, T1ρ could be used to identify whether metabolic differences are present in a psychiatric disorder with image-level resolution. These findings could then be followed up with MRS to identify specific metabolites that may be abnormal in regions-of-interest identified using T1ρ. Similarly, CEST imaging could be used to study differences in certain metabolite concentrations with high spatial resolution. Taken together, this potential for high resolution metabolite maps may allow for rapid, whole-brain screening for metabolic abnormalities in psychiatric illness and may also serve as a novel biomarker for assessing treatment response. Identifying such opportunities; and also identifying new ways to use chemical exchange imaging techniques is an area of ongoing research.

## Conclusion

Proton exchange is a novel target for measuring certain aspects of brain metabolic changes in vivo. Two brain imaging methods, CEST and T1ρ are sensitive to proton exchange and have been successfully implemented in clinical studies in human populations. However, their use to study psychiatric disorders has been minimal. CEST provides the ability to measure the concentration of specific molecules including a number of metabolic products and neurotransmitters that are thought to be relevant to psychiatric disorders. This selectivity suggests that CEST can be used as an complementary approaches to positron emission tomography (PET) or magnetic resonance spectroscopy (MRS) for certain experiments. T1ρ is less specific than CEST but is also more sensitive and can be carried out more quickly allowing better spatial and temporal resolution as well as for functional imaging. Therefore, T1ρ may be an alternative to PET and functional blood-oxygen-level-dependent (BOLD) contrast imaging. Continued improvement in these imaging techniques to shorten their acquisition time, sensitivity, and specificity may provide new insight into the neurobiology of these devastating diseases that to date have shown relatively subtle differences using MR imaging and have not yet provided a diagnostic test.

## Author Contributions

JS and MM were principally responsible for authoring the content of this review and are co-first authors. SS was responsible for the artistic creation of several figures and was substantially involved in the editing process. JX and NO provided technical knowledge regarding CEST and T1rho imaging and assisted with the editing process. DW assisted with editing and revising the manuscript. VM and JW are the senior researchers and provided feedback and were substantially involved in the editing and revision process. All authors contributed to the article and approved the submitted version.

## Funding

Several of the co-authors of this manuscript are funded by NIH (R01EB022019 and R01MH111578) to study the brain using T1ρ imaging. Some of the data shown in this manuscript was conducted on an MRI instrument funded by NIH (S10RR028821).

## Conflict of Interest

The authors declare that the research was conducted in the absence of any commercial or financial relationships that could be construed as a potential conflict of interest.
